# A non-invasive tool for detecting cervical cancer odor by trained scent dogs

**DOI:** 10.1186/s12885-016-2996-4

**Published:** 2017-01-26

**Authors:** Héctor Guerrero-Flores, Teresa Apresa-García, Ónix Garay-Villar, Alejandro Sánchez-Pérez, David Flores-Villegas, Artfy Bandera-Calderón, Raúl García-Palacios, Teresita Rojas-Sánchez, Pablo Romero-Morelos, Verónica Sánchez-Albor, Osvaldo Mata, Víctor Arana-Conejo, Jesús Badillo-Romero, Keiko Taniguchi, Daniel Marrero-Rodríguez, Mónica Mendoza-Rodríguez, Miriam Rodríguez-Esquivel, Víctor Huerta-Padilla, Andrea Martínez-Castillo, Irma Hernández-Gallardo, Ricardo López-Romero, Cindy Bandala, Juan Rosales-Guevara, Mauricio Salcedo

**Affiliations:** 1 Coordinación de Prevención y Atención a la Salud, Delegación Sur (Instituto Mexicano del Seguro Social) IMSS, Mexico City, Mexico; 2 Laboratorio de Oncología Genómica, Unidad de Investigación Médica en Enfermedades Oncológicas, UMAE Hospital de Oncología, CMN- SXXI-IMSS, Av. Cuauhtémoc 330, Col. Doctores, Del. Cuauhtémoc, 06720 Mexico City, Mexico; 3 Servicio de Braquiterapia, UMAE Hospital de Oncología, CMN-SXXI-IMSS, Mexico City, Mexico; 4 PEC de México, S.A. de C.V., Mexico City, Mexico; 5 Servicio de Oncología, Hospital General de Zona y de Medicina Familiar No. 5, IMSS, Taxco, Guerrero Mexico; 6 Clínica de la Mujer y Medicina Perinatal, Col. Roma Norte, Mexico City, Mexico; 7 Centro Colposcópico de Docencia e Investigación, A.C., Mexico, City, Mexico; 8 Departamento de Anatomía Patológica, Hospital General de Zona Troncoso, Mexico City, Mexico; 9 División de Neurociencias, Instituto Nacional de Rehabilitación (INR), Secretaría de Salud (S.S.), Mexico City, Mexico; 10 Facultad de Química, Universidad La Salle, Mexico City, Mexico

## Abstract

**Background:**

Cervical Cancer (CC) has become a public health concern of alarming proportions in many developing countries such as Mexico, particularly in low income sectors and marginalized regions. As such, an early detection is a key medical factor in improving not only their population’s quality of life but also its life expectancy. Interestingly, there has been an increase in the number of reports describing successful attempts at detecting cancer cells in human tissues or fluids using trained (sniffer) dogs. The great odor detection threshold exhibited by dogs is not unheard of. However, this represented a potential opportunity to develop an affordable, accessible, and non-invasive method for detection of CC.

**Methods:**

Using clicker training, a male beagle was trained to recognize CC odor. During training, fresh CC biopsies were used as a reference point. Other samples used included cervical smears on glass slides and medical surgical bandages used as intimate sanitary pads by CC patients. A double-blind procedure was exercised when testing the beagle’s ability to discriminate CC from control samples.

**Results:**

The beagle was proven able to detect CC-specific volatile organic compounds (VOC) contained in both fresh cervical smear samples and adsorbent material samples. Beagle’s success rate at detecting and discriminating CC and non-CC odors, as indicated by specificity and sensitivity values recorded during the experiment, stood at an overall high (>90%). CC-related VOC in adsorbent materials were detectable after only eight hours of use by CC patients.

**Conclusion:**

Present data suggests different applications for VOC from the uterine cervix to be used in the detection and diagnosis of CC. Furthermore, data supports the use of trained dogs as a viable, affordable, non-invasive and, therefore, highly relevant alternative method for detection of CC lesions. Additional benefits of this method include its quick turnaround time and ease of use while remaining highly accurate and robust.

## Background

Cervical cancer (CC) represents a serious public health concern worldwide among the female cancer spectrum. In Mexico, its incidence levels stand at an alarming 15.5% per year with a mortality rate of 12.8% [1].

Widely accepted in the scientific community, infection by Human Papillomavirus (HPV) is the main risk factor for CC development but its presence, however, is not sufficient for malignant transformation. In fact, a broad variety of co-factors and a significant number of molecular events exert influence in such process [2, 3]. Furthermore, the reprogramming of energy metabolism is now part of the hallmarks of cancer [4], undoubtedly comprised of important biological capabilities acquired during the multi-step development of human tumors and constituting an organizing principle for rationalizing the complexities of neoplastic disease.

Current standards for cancer diagnosis rely heavily on biopsy. In the case of CC, the standard extends to cytological and colposcopy procedures in addition to early detection of precursor lesions. With tests taking up to 1 month to return results, current diagnosis standards for cancer present an area of opportunity, particularly for developing countries and marginalized areas that face more severe issues such as the lack of proper medical and testing facilities.

Since the late 20th Century, reports of cancer detection by trained scent (sniffer) dogs have been on the rise, using different biological fluids such as urine, breath, blood, and stool with prompt results [5–8]. According to a recent report by Horvath [9], the specific odor of carcinoma plays an important role in the diagnosis and disease monitoring of cancer. In this context, various human cancers, such as breast, melanoma, lung, ovary, gastric, and prostate, have been considered for their evaluation by sniffer dogs as an accessible biological sample (direct or indirectly) [5–17].

The present work had as a goal the introduction and improvement of a non-invasive tool to aid in detection of cervical cancer, to test detection of CC-associated VOC by a sniffer dog, and testing different methods (both invasive and non-invasive) of harboring such compounds.

## Methods

Research met all ethical guidelines and practices, as overseen by the Comisión Nacional de Investigación Científica (Scientific Research National Committee) at the Instituto Mexicano del Seguro Social (Mexican Institute for Social Security, IMSS).

A total of 20 fresh biopsies, 50 CC smear samples, and 30 healthy cervical smears samples were employed in this research. All biopsy samples were collected from patients who attended Brachytherapy Service at the Oncology Hospital, CMN-SXXI-IMSS, in Mexico City. Normal cervices without HPV infection or precancerous lesions were also collected for use as control samples from routine gynecological examination patients at the Colposcopy Clinic. Additionally, a total of 70 patients affected by invasive CC used medical adsorbent surgical bandages as intimate sanitary pads to be used as samples. Commercial intimate feminine sanitary pads with nanomaterial that absorbs odor and fluids and with added scent, such as *Aloe vera* or *chamomile* were also admitted after approximately 8 h of use. Usage of surgical bandages by healthy women lasted 1 h, 8 h, 12 h, or 24 h.

Inclusion criteria for women participating in the study include: use of intimate vaginal scents and/or vaginal douches, being on any diet, alcohol or drug consumption, and age >20 years; women in their early or late phase menstrual period; women using oral contraceptives; one woman with diabetes; women who smoke; women with bacterial vaginosis; and three pregnant women of 3, 5, and 7 months. The majority of women resided in Mexico City, although others were from the state of México (1 h distance by car), Taxco, Guerrero (3 h distance by car), and Tuxtla Gutierrez, Chiapas (1.5 h distance by plane). Female participants, therefore, represent different ethnic groups from different environments with different lifestyles and diets which may have included a variety of spices and meats. All patients participating in this research provided prior approval and signed an informed consent form. Control samples, on the other hand, included, sink water, saline buffer, HPV vaccine (Gardasil [virus-like particles, VLP] 1 μg), plasmid DNA cloning of different fragments of the HPV genome, the CaSki cell line, aerosol tissue fixative, stem cell extracts (commercial products), white blood cells (WBC), red blood cells (RBC), earth/soil, exhaled breath, and sweaty finger.

Biopsies were sectioned and paraffin embedded. Hematoxylin and eosin (H&E) stained sections were analyzed to confirm tumor presence of at least 50% per sample, including squamous cervical carcinoma and adenocarcinomas. The first scrape of smear samples was obtained using a cervical brush for routine cytological examination while the remaining material was deposited in 50 ml Falcon tube. First round of H&E smears was always subjected to evaluation by a pathologist to determine and confirm cytological status. Because of the possibility of having mixed cell types in biopsies, expectation was to get fewer amounts of exfoliated cells.

All tests were carried out in a double-blind procedure. The two canine handlers participating in the study were experienced and present at all test times. They were responsible for recording results and did so wearing disposable polyethylene gloves at all times, exchanging them for a clean pair every two samples to avoid sample contamination.

Dog training, positive conditioning clicker method.

For the purpose of this research, one three-year-old male beagle was trained to identify and discriminate CC odor. To accomplish this, a 15-min training routine was conducted every morning, 5 days a week. During training, the beagle had his own cell to rest in, was allowed to play with other dogs without restriction and his regular diet composed of typical dog food remained the same. The beagle had no previous scientific experience but had been formerly trained for drug detection, however.

**Fig. 1:**
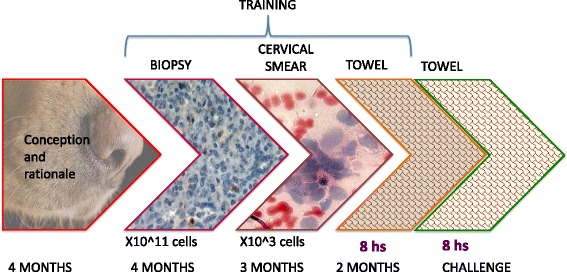
Study design. The figure summed up the time used for training. It is expected that the medical adsorbent surgical bandage or pad could harbor a range of 1 × 10^3–11 cells among other substances and molecules. The background in the narrows depicts tumor tissue (original amplification, 100×) and cervical smear (original amplification, 400×), Hematoxylin and eosin (H&E)-stained

Two groups of artifacts were used during training trials: 1) ten 20 cm cubic wood boxes with a 5 cm hole in the upper side, and 2) ten steel cylindrical containers measuring 15 cm of diameter and 30 cm high. The two groups were used interchangeably, but only one group would be used during a given trial. Artifacts were placed on the floor and arranged in a circle and about 50 cm apart from each other. Each artifact contained one sample. Samples were randomly arranged to include 9 healthy ones and 1 CC. Each trial comprised two runs. During the first run, the beagle was directed by the handlers to sniff all samples freely. The second time, the dog was directed to move towards the CC sample to instruct him which one was to be considered his target sample. This detection routine (Fig. 1) remained the same throughout research. Upon accurate display of desired behavior, handlers would indicate his success to the beagle using traditional clicker training techniques and then rewarded him with food. In this manner, the beagle learned to identify the odor of a fresh CC biopsy as the target sample and sit down in front of it to indicate his findings. The target sample became a referential marker for further experiments and considered as the “CC-scent.”

Upon completion of the training program, the beagle was presented with a new challenge: discrimination between healthy and cervical cancer smears. With the intention of properly assessing detection probability, CC cells were decreased in amount with different exposure methods, replacing CC biopsies with cytological smears, which contain a significantly lower amount of cells. Finally, the medical surgical bandages (with no added perfume scent) were introduced as samples during trials. Afterwards, a new series of 270 new surgical bandages were analyzed (group 1): 170 from healthy women as controls, and 100 from patients with CC (some patients with endometrial cancer were included in this group).

In order to avoid any pitfall molecule, present in the hospital- or clinic-rooms, all surgical bandages were used overnight in-home, and after that, each female subject deposited it into individual seal packaging bag and carried-out to the hospital or clinic to collect them. Under no circumstance were the bags opened in the hospital-or clinic-rooms and were always cleaned before use, to avoid any aromatic contamination.

False Alerts (FA) were called in and recorded every time the beagle marked a “healthy” sample as the target in a clear manner. Each of these occurrences was followed by proper, random repositioning of the target sample before running a new trial.

### Data analysis

Sensitivity is the primary parameter for measuring the dog’s success in marking a sample (bandage used by the oncologic patient) as target. Specificity is used instead to measure the dog’s performance in identifying patients without the disease [18].

Sensitivity and specificity values were calculated with 95% Confidence intervals (95%CI); positive and negative prognostic values were calculated by using Epidat v3.1 software. The gold standard was the biopsy (for cervical cancer sample) or cervical cytology (for healthy subjects). Positive and negative predictive values were adjusted to the CC prevalence by using Bayes theorem.

## Results

### Training to smell the fresh CC biopsy

A vital challenge to this research was training the beagle to identify CC’s volatile compounds. During his training, he worked only with a variety of CC biopsies of epidermoid and adenocarcinoma types. Healthy smears were only introduced later. The beagle’s first three trials exhibited various FAs. His training routine, explained earlier, endured for 4 months before he was able to fully and unequivocally identify the odor, showing no signs of hesitation when pointing target samples. Only then were cervical smears introduced into the sample pool, decreasing the amount cells both healthy and transformed in magnitude by several orders.

### The smears samples challenge

Smear samples, as it was to be expected, presented vaginal mucus and cervical cells from oncological patients’ blood. Interestingly, when introduced into the sample pool, only the first trial presented a FA. Again, only when the beagle succeeded in fully and unequivocally identifying the target sample as such were medical adsorbent materials introduced into the sample pool.

### The surgical bandages challenge

Like in smear samples, vaginal mucus and cervical cells were both present in all medical adsorbent material samples. However, traces of urine and blood were also detected. Surprisingly, neither represented an obstacle for the beagle to identify target samples.

### Flying solo, discerning CC scent volatile compounds

Research produced 873 test results from nearly 100 trials just from 97 cervical cancer smears and 776 from healthy women samples. Meanwhile, for surgical bandages, 495 test results from nearly 60 trials just from 55 oncological patients and 440 from healthy subjects.

Table 1 illustrates results obtained from trials where smears and adsorbent material samples were used. Sensitivity registered from both sample types was of 92,78 and 96,36%, respectively; corresponding specificity values stood at 99,1 and 99,55%; predictive values at 92,78 and 96,36%; and the negative predictive values at 99,1 and 99,55%. The false negative rate registered was notably lower in the case of adsorbent material samples (Table 1), suggesting this type of sample might be more efficient for medical applications to identify CC odor. Unexpectedly, the beagle displayed interest in all samples containing endometrial cancer cells (*n* = 10), risking proper identification of CC odor and suggesting similarities in their volatile compounds. Commercial intimate feminine sanitary pads with added scent also triggered a particular interest in the beagle. Exhaustive analysis indicates the pad’s material produced a false positive result. Hence, its inclusion as a capture method has been discarded. On the other hand, the beagle’s performance seemed unaltered by inert or chemical materials in control samples like vaginal douches, glass, cotton, lotions, cloned HPV DNA, the VLP vaccine, or live material such as blood or cells. Subjects’ places of origin and lifestyle conditions were not a significant indicator in FA results. Regarding the group 1 of samples analyzed, no FAs were registered for control samples while CC samples were marked correctly.

**Table 1:** Sensitivity and specificity measurements for cervical scrapes or medical adsorbent surgical bandages detected by a dog in patients with Cervical Cancer (CC)

	Sensitivity (95% CI*)	Specificity (95% CI)	PPV (95% CI)	NPV (95% CI)	PPV^α^	NPV^α^	FN
Scrapes	92,78% (87,12–98,45)	99,1% (98,37–9,83)	92,78% (87,1–98,45)	99,1% (98,37–99,83)	93,95%	98,91%	7
Surgical bandages	96,36% (90,51–100)	99,55% (98,8–100)	96,36% (90,51–100)	99,55% (98,8–100)	97,00%	99,45%	2

*Confidence Interval, *PPV* Positive Predictive Value, *NPV* Negative Predictive Value, ^α^Positive and negative predictive values were adjusted to the prevalence of Cervical cancer (CC) in Mexico (13.1% according to GLOBOCAN Project, reference 19), *FN* False Negative

## Discussion

Cervical Cancer is one of the most important health concerns among women in Mexico [19]. This research and, as such, the development of alternative methods for early and prompt detection of CC, represents a huge medical improvement for individuals and health institutions alike, potentially refocusing research efforts to other areas. In addition, it can provide great benefits to developing countries and marginalized regions exhibiting deficits in health services and facilities, which explain partially the high incidence of Cervical Cancer among their populations due to their extremely low income and widespread taboos and prejudices. Because of how damaging CC can be to female populations in these countries, addressing this health problem should be a priority.

**Fig. 2:**
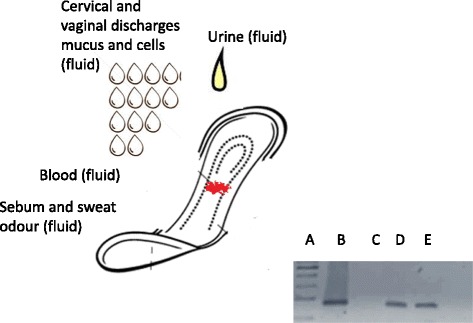
Medical adsorbent surgical bandages as “adsorber” of several human fluids for cancer detection. The pad could contain a mixture of cells (epithelial healthy and sick cells) and certain other fluids potentiating the cancer odor emitted by the patient. Also presented is the detection of mitochondrial DNA obtained from cells on the surface of the surgical bandage. Lane **a**) molecular weight marker 100 base pairs (bp) DNA ladder; Lane **b**) DNA positive control; Lane **c**) negative control; Lane **d**) amplicon (150 bp) from control subject without cervical lesion, and Lane **e**) from a Cervical cancer patient-affected (CC)

Inspired to contribute to global medical communities’ efforts, our team suggests the use of medical adsorbent surgical bandage as a fast, inexpensive, simple, safe, easy to use, non-invasive tool for capturing CC volatile compounds when used as a sanitary pad by patients. Its ability to collect several of the body’s metabolites such as scraped cells and mucus, urine drops, personal odor, sweat and sebum, which can be used to detect molecules related with cancer, in a manner that is acceptable for use by the patient makes it a viable solution (Fig. 2).

Different methods for harboring CC odor were also explored in the development of a strategy to discriminate between healthy and cancerous samples. Having as a premise dog’s ability to learn to identify cancer odor as specific organic compounds or “fingerprints,” our team concluded CC biopsies harboring mostly transformed cells (including other kinds of cells) represent the best starting point for training a dog. The amount of cells per sample supported our original theory that CC biopsies harbored enough information for a dog to identify and learn cancer odor. Furthermore, biopsies could harbor CC’s “fingerprint” without contaminant odors. Dog training procedures like the one employed in this research have been used before in successful detection of several cancer odors [5, 8, 12], supporting our original theory and conclusion.

Our team was challenged with reducing the number of cells from samples to measure any correlation between the number of cells contained within a sample and a dog’s ability to “pick up” the scent. Research results indicate there is none. Moreover, the beagle employed in this research displayed enormous ability in identifying CC odor regardless of the number of cells contained in the sample. Even more surprising, the beagle identified CC odor specifically rather than the subject or patient, discriminating and detecting specific CC samples. Research data, ultimately, supports the impressive ultra-fine olfactory system attributed to dogs [20, 21].

In present day, the scent of a number of diseases, including cancer, can be detected through the use of lab methods such as chromatography [8, 19–26]. There are great differences, however, between using chromatography and a dog, having a detection threshold of parts per billion (ppb) and parts per trillion (ppt) respectively [27]. Our research team thinks that first screenings (presumptive and rapid pre-screening) in isolated communities will become far more accessible if carried out by a trained dog rather than by sophisticated equipment. Obviously, this is presented as an alternative and caution is a must, as are additional studies on the subject. In general, after CC detection during the first screening, patients should be clinically evaluated. Our suggested method allows for minimization of time, resources, and money invested by the patient without sacrificing accuracy or robustness in test results. Based in current studies and data available, our research team suggests pad-based detection to be used only as a first screening test.

**Fig. 3:**
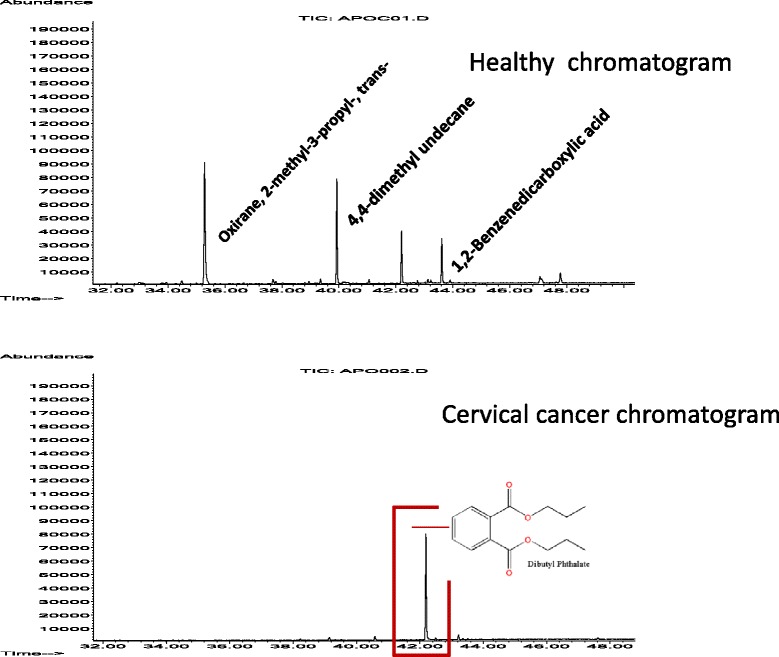
Comparison of gas chromatograph-mass spectrometry (volatile organic compounds) of adsorbent bandages used by healthy and Cervical Cancer-affected women. After usage of the adsorbent pads during 8 hours, these were subjected to an analysis by gas chromatography-mass spectrometry. The compounds were obtained by using the experimental conditions: hexane at 4°C with DB-column of 1.25 mm×60 m×0.25-μm and Helium gas carrier by employing Agilent gas chromatography-mass spectrometry equipment. The upper chromatogram represents a healthy woman, and the lower chromatogram from a Cervical Cancer-affected patient. The x-axis represents the time retention in minutes, while the y-axis the curve area. The graph is showing an example of mass spectrum of the following organic compounds: Oxirane; 2-methyl-3-propyl-trans; 5 H Tetrazol-5-amine; Eicosane and Dibutyl phthalate (DP) presented in the healthy women (Healthy chromatogram), while the 3 Ethyl-3-methyl heptane; 3,3 Dimethyl-1 [2 carboxyphenyl] triazine; and DP in the cancer patient (Cervical Cancer chromatogram), where a clear difference in DP concentrations between women was observed (vertical red rectangle). Chemical structure of the organic compounds is also showed

Chromatography from surgical bandages validates the presence of several volatile compounds in cervical cancer (Fig. 3). However, cervico-vaginal odor results only partially due to the cellular decomposition by microorganisms present in this area. Eight hours of use of adsorbent material produces a complex mixture of molecules from different physio and/or pathological fluids, sweat, urine, etc. Because of this, our research team considered that cancerous odor could be masked or even completely imperceptible. However, the beagle’s ability to identify CC odor seemed unaffected. In fact, the beagle was able to recognize specific substances related to cancer as memorized odors and even detect CC-scent from different types of samples. In other words, samples collected from both invasive and non-invasive methods work for presenting “cervical cancerous odor” to a trained sniffer dog.

Our findings are supported partially by a work in which a vaginal self-sampling at home showed efficacy and cost-effectiveness for HPV detection in CC screening [28]. Additionally, our team considers medical surgical bandages can become an environment-friendly tool for detecting cancer odor in the near future.

Finally, the beagle displayed ability to identify samples containing endometrial cancer. This could be explained partially by the biological capabilities acquired during multi-step development of the tumors [4] and that these two gynecological tumors could share common volatile compounds. The latter is supported by a report by Horvath et al. (2008) which describes a dog trained to detect ovarian cancer was also able to detect other types of cancer, ultimately resulting in a potential drop in specificity values [9]. Our research team is currently performing additional studies to provide clarity to the subject. If our theory is correct, the applications for surgical bandages as non-invasive tool could broaden to include the detection of endometrial cancer and other tumors like ovary and breast cancers. Very recently, we have determined that sanitary pads are also able to collect VOC related to ovary cancer samples, as well as from breast samples. We are also employing a mask to collect volatile compounds from exhaled breath for several cancers of the upper aero-digestive tract (data not shown).

## Conclusions

Our research team is convinced that the trainer-dog partnership and the use of surgical bandages as the means to collect samples, both described in this research, are viable alternative tools for exploring and detecting cervical cancerous odor, even more when paired together. The benefits of these tools include inexpensiveness, accuracy, ease of use, non-invasiveness, and high sensitivity and specificity. Applications for these tools extend to providing much needed medical attention for women from cultural backgrounds imposing several prohibitions, deep-rooted cultural taboos, or lack of health coverage. Additionally, the use of a trained dog for screening facilitates prevention campaigns in areas of difficult access, saving money, labor, and the loss of lives due to late diagnosis. Finally, surgical bandages could also be used to detect endometrial cancer and potentially other tumors like ovary and breast cancer.

## Abbreviations

CC: Cervical cancer
CI: Confidence Intervals
DP: Dibutyl phthalate
FA: False Alerts
H&E: Hematoxylin and eosin
HPV: Human Papillomavirus
IMSS: Instituto Mexicano del Seguro Social
ppb: Parts per billion
ppt: Parts per trillion
RBC: Red blood cells
VLP: Virus-like particles
VOC: Volatile organic compounds
WBC: White blood cells
